# Editorial: High Frequency Brain Signals: From Basic Research to Clinical Application

**DOI:** 10.3389/fnhum.2022.872478

**Published:** 2022-03-30

**Authors:** Jing Xiang, Ryouhei Ishii, Xiaofeng Yang

**Affiliations:** ^1^Department of Pediatrics and Neurology, Cincinnati Children's Hospital Medical Center, Cincinnati, OH, United States; ^2^Graduate School of Comprehensive Rehabilitation, Osaka Prefecture University, Habikino, Japan; ^3^Bioland Laboratory, Guangzhou Regenerative Medicine and Health, Guangzhou, China

**Keywords:** high frequency brain signal, high frequency oscillation, ripple, fast ripples, high gamma oscillation

High frequency brain signals (HFBS) can be divided into endogenous (spontaneous) HFBS and evoked HFBS. Endogenous HFBS are preliminarily detected in epilepsy patients. Endogenous HFBS in epilepsy include high frequency oscillations (HFOs), very high frequency oscillations (VHFOs), ripples, and fast ripples. Elicited or evoked HFBS are typically called high gamma (oscillations), which are functional activation and have been identified in the somatosensory, motor, visual, auditory, language and several other nerve systems. HFBS are typically recorded with invasive recordings, which include intracranial electroencephalography (iEEG), electrocorticography (ECoG) and stereoelectroencephalography (SEEG). Recent advances in magnetoencephalography (MEG) and scalp electroencephalography (EEG) have shown promising results for non-invasive detection of HFBS. Invasive recordings provide convincing data, although its applications are limited to accessible brain areas (low spatial sampling). Modern MEG can detect HFBS from the entire brain, which opens a new avenue for non-invasive detection and localization of HFBS.

Since epilepsy is one of the world's most common neurological disorders, it is important to find new biomarkers in epilepsy. Birk et al. showed that HFOs were significantly increased during ictal periods and significantly decreased during postictal periods compared to the ictal segment in a chronic focal epilepsy model in rats. Ictal ripples and fast ripples were significantly higher in severe seizures than in mild seizures. The results of this study indicate that HFOs can be used as biomarkers for measuring seizure severity in epilepsy. Ramon and Holmes showed that EEG ripples were markers of abnormally increased cortical excitability. Phase cone clustering patterns in EEG ripple bands were potential tools that could assist in localizing the epileptogenic zone (EZ).

Despite epilepsy being one of the most common neurologic disorders seen in children, there is no clear consensus regarding whether to treat or not to treat epileptiform discharges after a first unprovoked seizure. El Shakankiry and Arnold addressed the question by analyzing the coexistence of ripples/ HFOs with interictal epileptiform discharges in routinely acquired scalp EEG. The results showed that including analysis of HFOs in routine EEG interpretation could guide the decision to either start or discontinue anti-seizure medication. Yan et al. showed that the energy of HFOs could distinguish physiological HFOs from pathological ones more accurately than frequency. On scalp EEG, gamma oscillations could better detect susceptibility to epilepsy than ripple and FR oscillations. HFOs could trigger spasms. Of note, the analysis of average HFO energy could be used as a predictor of the effectiveness of epilepsy treatment in children.

One limitation in clinical applications of HFOs is that there is a significant inter-individual variation among patients. Qi et al. showed that the relative strength of HFO, especially fast ripples, was a promising effective biomarker for identifying the EZ with minimum individual variation. Clinical resection of the brain areas generating HFOs with the highest relative strength could lead to a favorable seizure outcome. Li et al. showed that the combination of PET-MRI and HFOs provided significantly more information than each modality alone for precise localization of EZs. The periphery of the lesion marked by neuroimaging might be epileptic, but not every lesion contributed to seizures. Therefore, approaches in multimodality could detect EZ more accurately, and HFO analysis helped in defining real epileptic areas.

Multi-frequency analysis of HFBS is important for basic research and clinical applications. Ren et al. showed that both ripples and fast ripples superimposed more frequently on slow waves in EZ than in non-EZ. Although ripples preferred to occur on the down state of slow waves in both two groups, ripples in EZ tended to be closer to the down-state peak of slow wave than in non-EZ. Slow wave-containing ripples in EZ had a steeper slope and wider distribution ratio than those in the non-EZ. But for slow wave-containing FR, only a steeper slope could be observed. A combination of HFOs and slow wave in EZ and non-EZ from refractory focal epilepsy could improve surgical outcomes. Sun et al. showed that the termination of absence seizures was associated with a dynamic neuromagnetic process. Frequency-dependent changes in the functional connection could be observed during seizure termination. Neuromagnetic activity in different frequency bands might play different roles in the pathophysiological mechanism during absence seizures.

Localization of HFBS provides crucial information for clinical treatment of epilepsy. Xu et al. showed the usage of HFOs for radiofrequency thermocoagulation (RFTC) for surgical treatment of drug-resistant focal epilepsy. The results of this study showed that RFTC targeting at brain areas generating ictal high-frequency-discharge, fast-rhythm, and low-voltage could result in favorable surgical outcomes. Wang et al. showed that fast ripples could be extensively detected in tuberous sclerosis complex patients using SEEG. Interestingly, high fast ripple rate contacts were mostly localized in the junction region of the epileptogenic tuber, which could aid in the localization of epileptogenic tubers for surgical treatment.

HFBS can be detected in patients with persistent postural-perceptual dizziness (PPPD). Jiang et al. showed that the frontal cortex generated aberrant activities in 1–4, 80–250, and 250–500 Hz in PPPD patients compared with healthy controls. The finding indicated that alterations in the frontal cortex played a critical role in the pathophysiological mechanism of PPPD.

## Author Contributions

All authors listed have made a substantial, direct and intellectual contribution to the work and approved it for publication.

## Conflict of Interest

The authors declare that the research was conducted in the absence of any commercial or financial relationships that could be construed as a potential conflict of interest.

## Publisher's Note

All claims expressed in this article are solely those of the authors and do not necessarily represent those of their affiliated organizations, or those of the publisher, the editors and the reviewers. Any product that may be evaluated in this article, or claim that may be made by its manufacturer, is not guaranteed or endorsed by the publisher.

